# Ionic Liquid-Assisted Thermal Evaporation of Bimetallic Ag–Au Nanoparticle Films as a Highly Reproducible SERS Substrate for Sensitive Nanoplastic Detection in Complex Environments

**DOI:** 10.1021/acs.analchem.3c04442

**Published:** 2024-03-07

**Authors:** Rafael V. Carreón, Orlando Cortázar-Martínez, Ana G. Rodríguez-Hernández, Laura E. Serrano de la Rosa, José Juan Gervacio-Arciniega, Siva Kumar Krishnan

**Affiliations:** † Facultad de Ciencias Físico Matemáticas, Benemérita Universidad Autónoma de Puebla, Av. San Claudio y Av. 18 sur., Puebla, Pue. C. P. 72570, México; ‡ CINVESTAV-Unidad Querétaro, Libramiento Norponiente No. 2000, Real de Juriquilla, Querétaro, Qro 76230, México; § CONAHCyT-Centro de Nanociencias and Nanotecnología, Universidad Nacional Autónoma de México, Km 107 Carretera Tijuana-Ensenada Apdo Postal 14, C. P. 22800 Ensenada, B.C., México; ∥ Instituto de Física, Benemérita Universidad Autónoma de Puebla, Apdo. Postal J-48, Puebla, Pue. 72570, México; ⊥ CONAHCyT- Facultad de Ciencias Físico Matemáticas, Benemérita Universidad Autónoma de Puebla, Apdo. Postal J-48, Puebla 72570, México; # CONAHCyT-Instituto de Física, Benemérita Universidad Autónoma de Puebla, Apdo. Postal J-48, Puebla, Pue. 72570, México

## Abstract

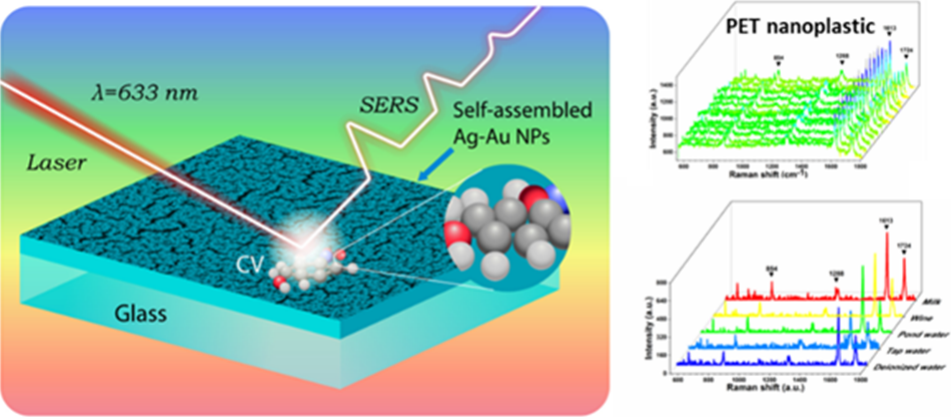

Nanoplastic particles are emerging as an important class of environmental pollutants in the atmosphere that have adverse effects on our ecosystems and human health. While many methods have been developed to quantitatively detect nanoplastics; however, sensitive detection at low concentrations in a complex environment remains elusive. Herein, we demonstrate a greener method to fabricate a surface-enhanced Raman spectroscopy (SERS) substrate consisting of self-assembled plasmonic Ag–Au bimetallic nanoparticle (NP) films for quantitative SERS detection of nanoplastics in complex media. The self-assembly of Ag–Au bimetallic NPs was achieved through thermal evaporation onto a vapor-phase compatible ionic liquid based on deep eutectic solvent over the growth substrate. The finite-difference time-domain simulation revealed that the localized field enhancement is strong in the gaps, which generate uniform SERS “hotspots” in the obtained substrate. Benefiting from highly accessible SERS “hotspots” at the gaps, the SERS substrate exhibits excellent sensitivity for detecting crystal violet with a limit of detection (LOD) as low as 10^–14^ M and excellent reproducibility (RSD of 5.8%). The SERS substrate is capable of detecting PET nanoplastics with LOD as low as 1 μg/mL and about 100 μg/mL in real samples such as tap water, lake water, diluted milk, and wine. Moreover, we also validated the feasibility of the designed SERS substrate for the practical detection of PET nanoplastics collected from commercial drinking water bottles, and it showed great potential applications for sensitive detection in actual environments.

## Introduction

Owing to the large-scale production of plastics, an increasing amount of mismanaged plastic waste is accumulated and degraded in the environment, causing severe environmental pollution, which is an emerging concern worldwide for various ecosystems and human health.^1,2^ For instance, it was estimated that about 4.8–12.7 million metric tons of plastic waste are mixed in the world’s oceans each year and will be expected to increase by about 12 billion metric tons by 2050,^3^ which may cause a severe environmental pollution crisis.^1^ It is explicitly assumed that the vast majority of environmental microplastic particles (<5 mm) were generated through the natural fragmentation of larger plastic debris by photochemical or biotic degradation processes that have been discharged into aquatic environments.^4^ However, pollution caused by the smaller plastic particles (<100 nm), typically known as nanoplastics, and their impact on the environment and human health is poorly understood.^5^

Traditional methods to detect micro/nanoplastics are mainly based on the microscopy technique or spectroscopy-based techniques, such as scanning or transmission electron microscopies,^6^ flow cytometry,^7^ Impedance spectroscopy,^8^ infrared spectroscopy.^9^ However, these methods present significant challenges to detect nanoplastics because of their smaller particle size, and lack of sensitivity in dilute concentrations.^10^ Surface-enhanced Raman Scattering (SERS) based on plasmonic nanoparticles (NPs) has emerged as the most powerful and reliable analytical tool due to its high spatial resolution and multiplexing capability and requires no complicated sample preparation.^11^ Especially, the SERS sensors constructed on plasmonic nanoparticles such as silver (Ag), gold (Au), and Ag–Au bimetallic NPs are highly efficient because of their tunable localized surface-plasmon resonance (LSPR) properties and strong electromagnetic (EM) field distributions around the NPs.^12,13^ The SERS enhancement factors (EFs) in the plasmonic substrate critically rely on NP size, surface structure, interparticle nanometric gaps, sharp tips or edges of noble metal NPs, where the electric field is much stronger, typically called “hotspots”.^12^ Although all these factors contribute to enhancing the SERS EFs, the creation of a three-dimensional (3D) bimetallic nanostructure and uniform subnanometric gaps could amplify the SERS signals to several orders of magnitude, which enables the detection of analyte molecules at a single molecule level.^14^

The development of a 3D structure on a substrate through self-assembly processes is an effective strategy to generate “hotspots” and improve the SERS sensitivity.^15,16^ Self-assembled plasmonic nanostructures were prepared commonly through either electron-beam lithography or photolithography techniques,^17^ nanoimprint lithography,^18^ or conventional colloidal self-assembly process.^19^ However, these methods present a significant challenge and are costly.^20^ Alternatively, the vacuum thermal evaporation technique has been considered a low-cost and highly efficient method to grow high-purity metallic thin films on a solid substrate.^21^ However, the formation of nanoparticles and porous structure is difficult with the thermal evaporation technique. Thus, various organic solvents or liquids have been used to deposit metallic/bimetallic NPs onto surfaces, effectively controlling their morphology and self-assembly structures.^22^ At present, deep eutectic solvents (DESs) are emerging as a new type of solvents, which are relatively nontoxic and biodegradable compounds. DESs are easily obtained by mixing hydrogen bond donors and acceptors.^23^ The DES exhibits a high density (1.0 and 1.3 g cm^–3^),^24^ and low vapor pressure at room temperature,^25^ and thermal stability, which makes it compatible with vacuum conditions.^26^ In addition, an extended hydrogen-bonded network in the DES can promote the self-assembly of the deposited nanoparticles over the DES surface.^27^ However, it is challenging to integrate DES with the thermal evaporation process to fabricate self-assembled metallic NPs.

In this work, we develop a simple and efficient method for fabricating self-assembly of Ag–Au bimetallic NP films with tunable SERS “hotspots” for sensitive SERS detection of nanoplastics. The controlled self-assembly of the Ag–Au NPs using a simple vacuum thermal evaporation technique over “greener” ionic liquids based on DESs, facilitates the self-assembly of Ag–Au NPs. By optimizing deposition pressure and ratio between Ag/Au, we could easily achieve a uniform self-assembly structure of Ag–Au NP films with tunable shape and size of the gaps. The SERS substrate made with the self-assembled Ag–Au NP film exhibits a limit of detection (LOD) of 10^–14^ M, excellent reproducibility and stability for the detection of crystal violet (CV) as a probe molecule. Finally, we show that the SERS substrate could detect trace amounts of PET nanoplastic particles with an excellent sensitivity of PET concentration of 100 μg/mL in commonly used environmental and food samples.

## Experimental Section

### Preparation of DES

DES was obtained by following the procedure reported elsewhere.^27^ DES was formed by mixing choline chloride (HOC_2_H_4_N[CH_3_]_3_Cl; ≥ 98%; Sigma-Aldrich) and urea (NH_2_CONH_2_, 99.0%, Sigma-Aldrich) in a molar ratio of 1:2. Prior to mixing these both components, the choline chloride (ChCl) was first dried at 90 °C in an oven for one h to ensure that the ChCl was completely dry. Then ChCl and urea were mixed, and the mixture was sealed and heated in an oven at 90 °C until a transparent and homogeneous liquid was formed. The viscous liquid was then cooled to room temperature and stored for later use.

### Fabrication of SERS Substrate by Self-Assembly of Ag–Au NPs Using Thermal Evaporation Technique

For the preparation of Ag–Au NP films, the 2.5 × 2.5 cm^2^ glass substrates were cleaned and then the DES was dropped evenly over the glass substrates with a uniform thickness, and were mounted inside the vacuum chamber down to the tungsten filament. The deposition process was carried out in a vacuum chamber by introducing a certain amount of Ag–Au metallic wire (33 mg of Ag and 31 mg of Au, 99, 99% purity Ted Pella, Inc.) inside the tungsten filament and then thermally evaporated by supplying a current of 4 A to the tungsten filament. After that, the deposited Ag–Au NP films were gently covered with another clean glass slide on the surface, and then the sandwich formed with the glass slides was kept for a few minutes so that the film adhered better to the clean glass slide. Finally, the sample was immersed in deionized (DI) water for 5 min to dissolve the DES to obtain the self-assembled Ag–Au bimetallic NP film. The Ag/Au ratio was varied to (1:4, 1:2, 1:1, 2:1, 4:1 wt %) to obtain different Ag/Au content in the resultant films.

### Finite-Difference Time-Domain (FDTD) Simulation

The FDTD optical simulations were performed using FDTD solutions software (Lumerical Inc., Canada version). The dielectric constants of gold and silver, obtained from the CRC Handbook of Chemistry and Physics, are provided in the material database of the software; for homogeneous Ag–Au alloy spheres, the dielectric constants can be calculated from the earlier report.^28^ The experimental simulation region was set to 600 × 600 nm^2^ with a mesh accuracy of 1.0 nm in the areas around nanoparticles. A plane wave polarized light with a wavelength of 633 nm was used along the *z*-axis.

### Characterization Techniques

Field-emission scanning electron microscopy (FE-SEM) images were obtained using a JEOL-JSM7401F field-emission scanning electron microscope operating at 20 kV. Atomic force microscopy (AFM) topological images were taken by an XE-7 from Park Systems. The UV–vis absorption spectra of the films were recorded using an Agilent 8453 UV–vis spectrophotometer (Agilent spectrophotometer) in the spectral range of 300–1100 nm. X-ray diffraction (XRD) spectra of the samples were collected using a Rigaku Ultima IV X-ray diffractometer, using Cu Kα radiation (λ = 1.0654 Å). The XPS spectra were collected using an XPS instrument assembled by Intercovamex (Morelos, Mexico) with a dual X-ray source of aluminum Kα1 (*h*ν = 1486.7 eV) (XR4, from ThermoFisher, East Grinstead, UK) and a 7-channeltron hemispherical spectrometer (Alpha110, also from ThermoFisher). The XPS peak fitting analysis was done using the software AAnalyzer, which contains advanced tools and analysis methods. SERS spectra of the samples were collected from a Bruker (SENTERRA) Raman spectrometer using an excitation laser wavelength of 633 nm.

### SERS Measurements

The SERS activity of different self-assembled Ag–Au NP films was examined using CV and Rhodamine 6G (R6G) as a probe molecule. Specifically, 25 μL (10^–6^ M) of an aqueous solution of CV and R6G were dropped over the Ag–Au NP film substrates and allowed to dry at room temperature for 3 h. After that, SERS spectra were collated laser excitation using a 633 nm, laser power of 1.6 mW. The diameter of the exciting laser spot was fixed to 4.7 μm under a 50× lens, and the signal acquisition time was fixed to 2 s for 3 cycles.

### SERS Detection of PET Nanoplastic Particles

The PET nanoplastics with sizes ranging between 50 and 300 nm were prepared by following the earlier work.^29^ For SERS detection, first the PET nanoplastics (size 50–300 nm) of various concentration was dispersed in DI water under sonification for 1 h until it disperses completely. Then, 25 μL of PET nanoplastic particle dispersion was deposited onto the prepared Ag–Au substrate through drop-casting, and then the substrate was allowed to dry at ambient conditions for 6 h. After that, the substrates were taken for SERS analysis.

### SERS Detection in Real Samples

For real-sample analysis, the tap water and river water were collected from the lab and nearby lake. The wine and milk were purchased from a local store nearby and diluted in DI (1 mL of samples in 10 mL of DI water). For SERS detection, 1 mL (100 μg/mL) of PET nanoplastic solution was spiked in 10 mL of DI water, tap water, river water, diluted milk, and wine samples. Then, 25 μL of solution was drop-casted over Ag–Au NP substrate and allowed to dry naturally under ambient conditions prior to the SERS measurements.

## Results and Discussion

### Fabrication and Characterization of SERS-Active Self-Assembled Ag–Au NP Film

**Scheme 1:**
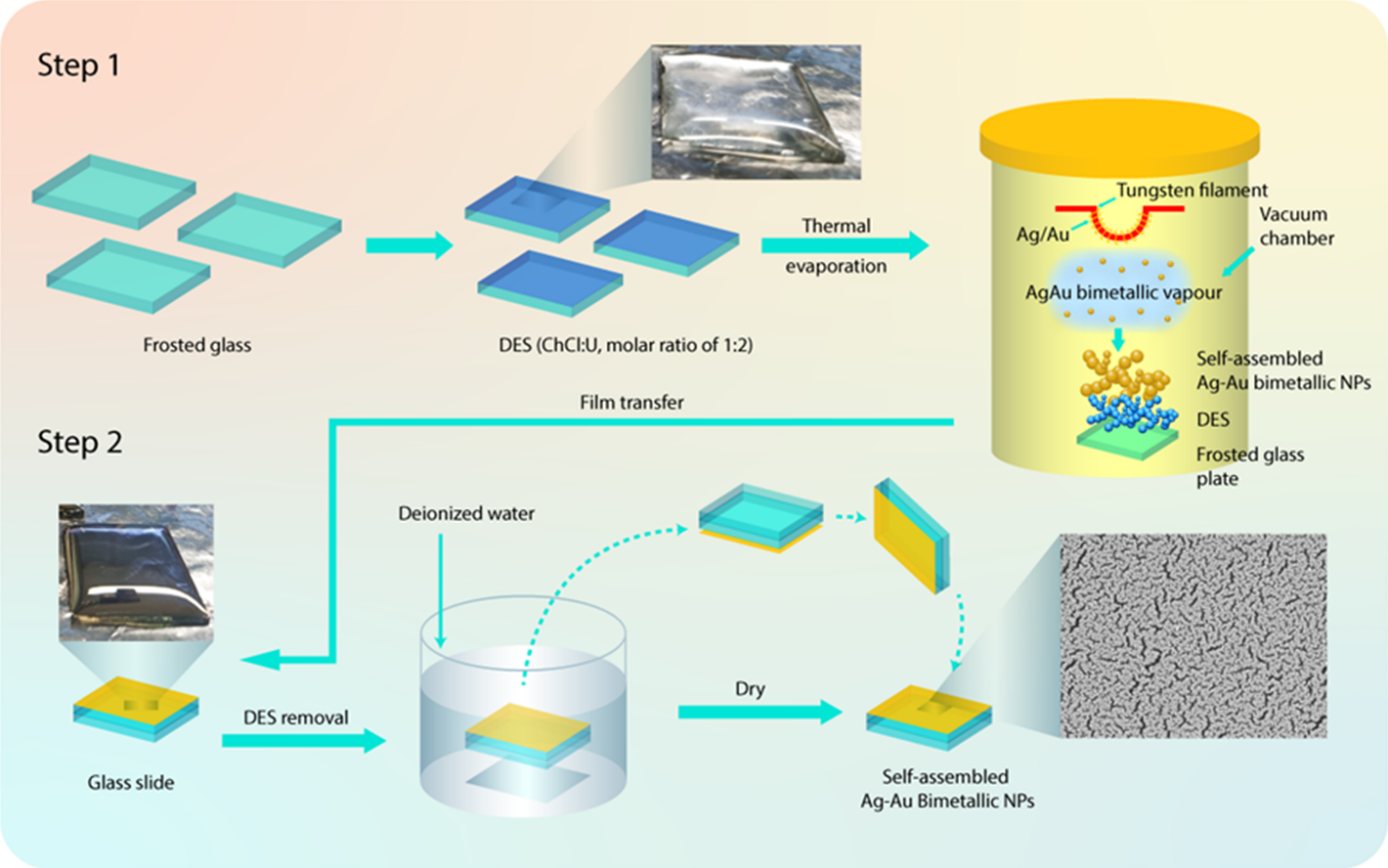
Schematic of the Vacuum Thermal Evaporation Process over Deep Eutectic Solvent (DESs) Coated over Glass Substrate for Controlled Self-Assembly of Ag–Au NP Film-Based SERS Substrate

The experimental procedure of a simple, DES-based ionic-liquid-driven self-assembly process of bimetallic Ag–Au NP films with controllable particle size and interparticle gaps is shown in Scheme 1. As can be seen in Scheme1, the self-assembly of Ag–Au NP films was achieved through vacuum evaporation of Ag–Au wire onto the DES surface, where DES served both as an efficient liquid substrate and soft template to the stabilizing agent. When increasing the deposition pressure in the vacuum thermal evaporation process, the coalescence among the metal ion vapors could induce the formation of larger nanoparticle assemblies with different shapes onto the liquid interface.^27^ A major advantage of utilizing DES is that there is no requirement for additional ligands or stabilizing agents and relatively low cost of the DES components (ChCl and urea) compared to the conventional ILs,^30,31^ and no requirement for sophisticated equipment for the thermal evaporation process. Thus, the proposed technique is highly cost-effective in the preparation of metallic/bimetallic NP films with a controlled self-assembly structure.

**Figure 1:**
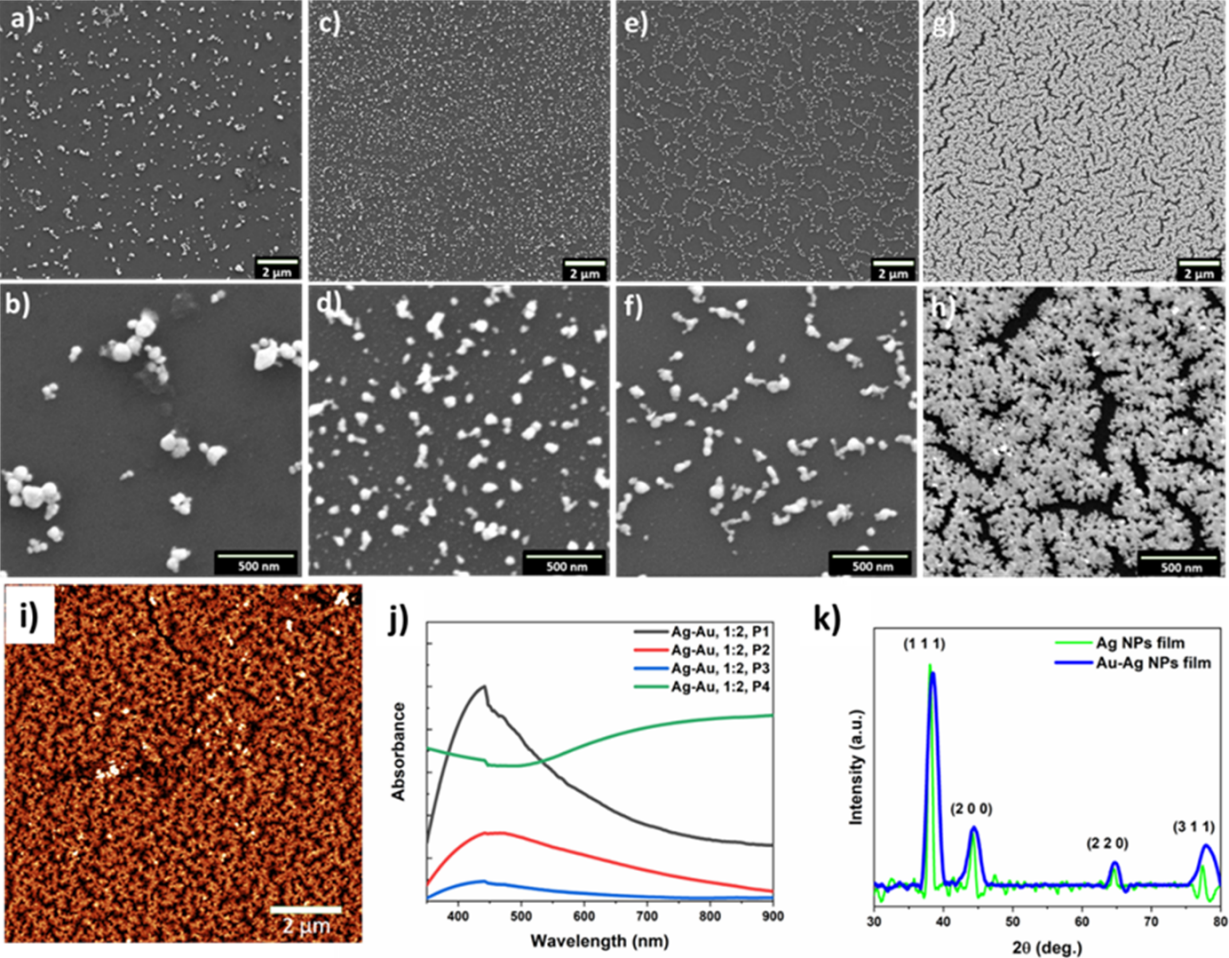
Representative low and high magnification FE-SEM images of self-assembled Ag–Au NP films under thermal evaporation at different pressures (a, b) 1 × 10^–2^ mbar, (c, d) 1 × 10^–3^ mbar, (e, f) 1 × 10^–4^ mbar, and (g, h) 2 × 10^–4^ mbar, respectively. (i) An AFM image of the self-assembled Ag–Au NP films obtained at 2 × 10^–4^ mbar, (j) UV–vis absorption spectra of Ag–Au NP films obtained at different pressures, and (k) XRD pattern of the Ag–Au NP films obtained at 2 × 10^–4^ mbar. For comparison, the XRD pattern of the bare Ag NP film was included (green).

Figure 1 shows the FE-SEM images of as-deposited Ag–Au NP films obtained at varying pressures of 10^–2^, 10^–3^, 1 × 10^–4^, and 2 × 10^–4^ mbar, which were denoted as Ag–Au (1:2) P1, P2, P3, and P4, respectively. As can be seen from Figure1a–d, the SEM images revealed that the Ag–Au films obtained at low pressures are mainly composed of nearly spherical nanoparticles that are distributed throughout the substrate. Upon increasing the deposition pressure to 1 × 10^–4^ mbar, the deposited films consist of smaller spherical Ag–Au NPs that are uniformly self-assembled into one-dimensional (1D) chain-like networks (Figure 1e,f)**.** However, further increasing the deposition pressure to 2 × 10^–4^ mbar, the obtained films were mainly composed of dendritic-shaped Ag–Au nanostructures that are self-assembled into closely packed structures with uniform nanometric gaps, as shown in Figures 1g,h and S1. The AFM topographical image of the Ag–Au (1:2) P4 sample (Figure 1i), exhibits highly uniform Ag–Au nanostructures that are densely packed structures, which further confirms the formation of well defined gaps between each dendritic nanostructure observed. However, no such self-assembly of NPs was obtained without introducing the DES, instead a smooth Au film was formed (Figure S2). Moreover, by changing the type DES composition, the morphology of the Ag-au NPs is easily tailored (Figure S3). The above results suggest that for achieving self-assembly of Ag–Au NPs over the DES surface, the deposition pressures need to be high. The UV–vis absorption spectra (Figure 1j) showed a broad absorption band appears in the 350–850 nm, suggesting LSPR peaks of bimetallic Ag–Au alloy films with porous network.^32^ In addition, the XRD pattern of Ag–Au films (Figure 1k) shows a slight shift in the characteristic diffraction peaks of (111), (200), (220), and (311) planes along with broadening of the peaks compared to the bare Ag NPs film, indicating the formation of Ag–Au alloy NPs.^33^

**Figure 2:**
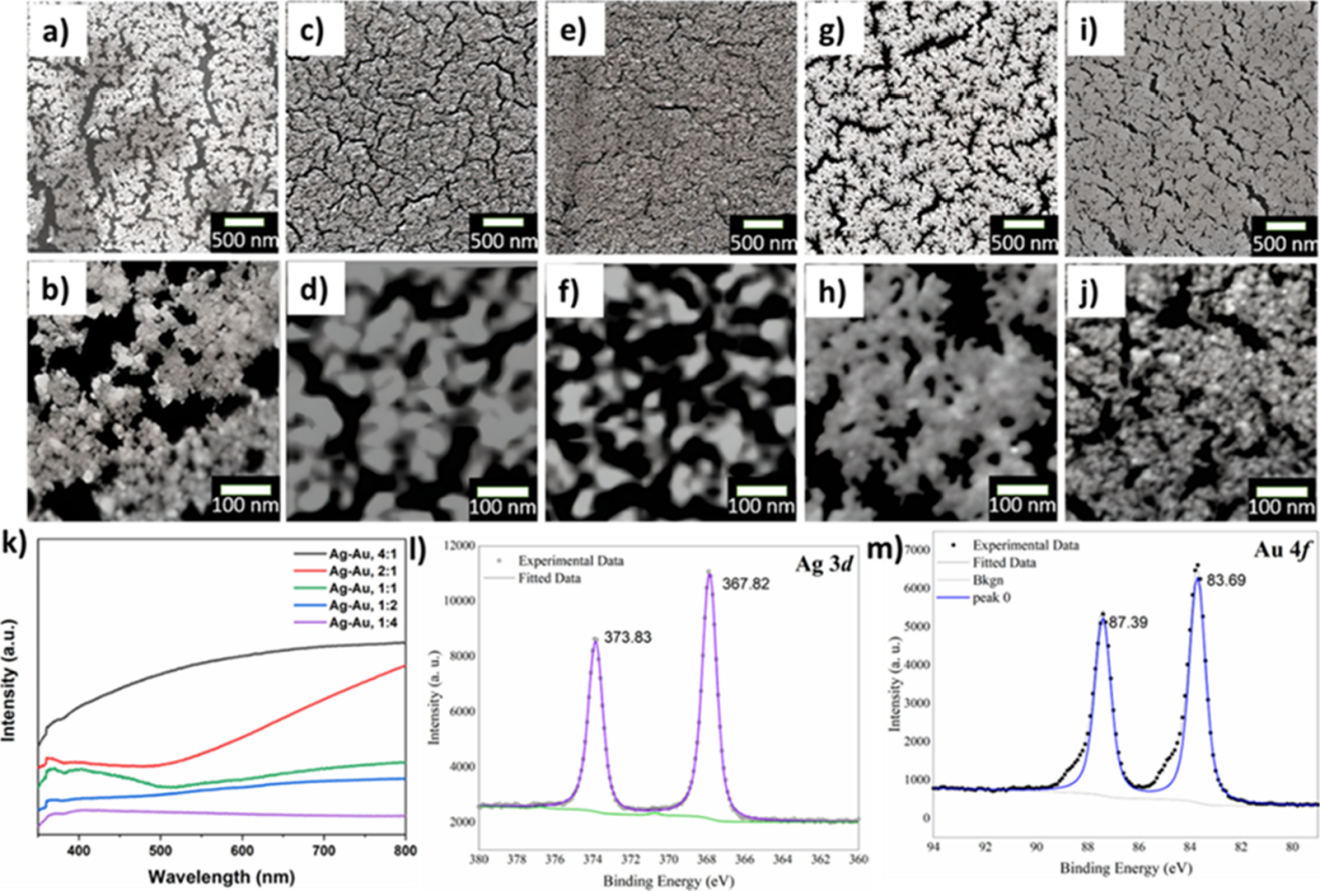
Low and high magnification SEM images of self-assembled Ag–Au NP films with different Ag/Au ratios (wt %). (a, b) 1:4, (c, d) 1:2, (e, f)1:1, (g, h) 2:1, and (i, j) 4:1, respectively. (k) UV–vis absorption spectra of Ag–Au films with different Ag/Au compositions. (l, m) high-resolution XPS spectra of Ag 3d and Au 4f core levels.

To modulate the morphology and surface structure of the substrate, the amount of Ag/Au was optimized. Figure 2a–j shows the low magnification SEM images of Ag/Au NP films obtained by varying Ag/Au content. The SEM images revealed the formation of uniform Ag–Au NP films with different self-assembled structures upon changing the weight ratio between Ag/Au. The films obtained with Ag/Au weight ratio of 1:2 wt % yield highly uniform dendritic-like nanostructures. However, upon increasing the Au content (Ag/Au ratio of 1:4) and Ag (Ag/Au ratio of 4:1), the formed films were highly porous structures with inhomogeneous surface morphologies. The energy-dispersive spectroscopy (EDS)-elemental mapping analysis of three different Ag–Au ratios (Figure S4) further confirmed that both Ag–Au elements are uniformly distributed, indicating the formation of Ag–Au bimetallic alloy NP film.

The UV–vis absorption spectra of Ag–Au NP films with varying Ag/Au content (Figure 2k) revealed that the intensity of the SPR peak position keeps decreasing and the broad absorption peaks are further red-shifted toward the near-infrared (NIR) region when increasing the Au content, which could be because of the change in their morphology and pore size.^34^ The high-resolution X-ray photoelectron spectroscopy (XPS) spectrum of Ag 3d core levels (Figure 2l) exhibits two binding energy (BE) peaks at 367.82 and 373.832 eV originating from Ag 3d_5/2_ and Ag 3d _3/2_ orbitals of metallic Ag^0^.^35^ On the other hand, the high-resolution XPS spectrum of Au 4f core levels (Figure 2m) exhibits two BE peaks at 83.69 and 87.39 eV that are very close to the Au 4f_7/2_ and Au 4f_5/2_ orbitals of metallic Au^0^.^36^ Importantly, the BE peak of Au 4f_7/2_ is found to slightly shift toward higher BE values in comparison with bare Au (Au 4f_7/2_ = 83.42 eV). Such shits of Ag 3d_5/2_ and Au 4f_7/2_ BE peaks could be due to the formation of Ag–Au bimetallic alloy nanostructures, since Au, being the most electronegative metal, gains electron density when the formation Ag–Au alloy NPs.^37^

**Figure 3:**
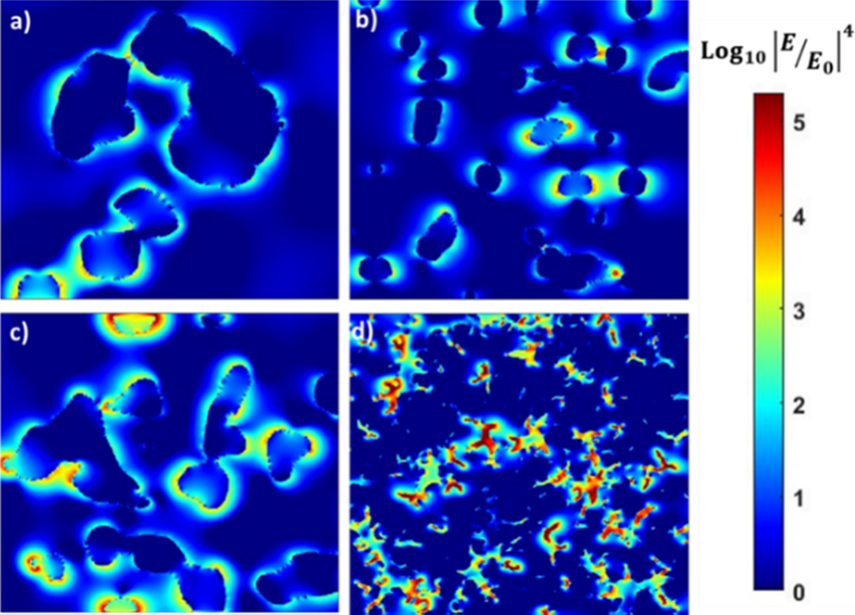
FDTD simulation of models of Ag–Au NP films obtained at different pressures (a) Ag–Au (1:2) P1, (b) Ag–Au (1:2)) P2, (c) Ag–Au (1:2) P3, and (d) Ag–Au (1:2) P4, respectively.

To gain more insights into the near field local EM field distribution and formation of SERS “Hotspots” in the Ag–Au NP films, finite-difference time-dominance (FDTD) simulations were carried out (Figure 3). The highest EM field intensities of the “hotspots” at the gaps were observed for dendritic-like Ag–Au NPs under an excitation wavelength of 633 nm laser light. It is well reported that the overall theoretical SERS EFs were approximately proportional to the fourth power of the enhancement of the local electric field by the following relation:
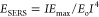
1where *E*_max_ and *E*_0_ are the maximum and an incident local field, respectively.^38^ The maximum value of *IE*_max_/*E*_o_*I*^4^ can be observed for the dendritic-like Ag–Au NPs network, which corresponds to an EF in the order of 6 × 10.^6^

### SERS Performance of Self-Assembled Ag–Au NP Films

**Figure 4:**
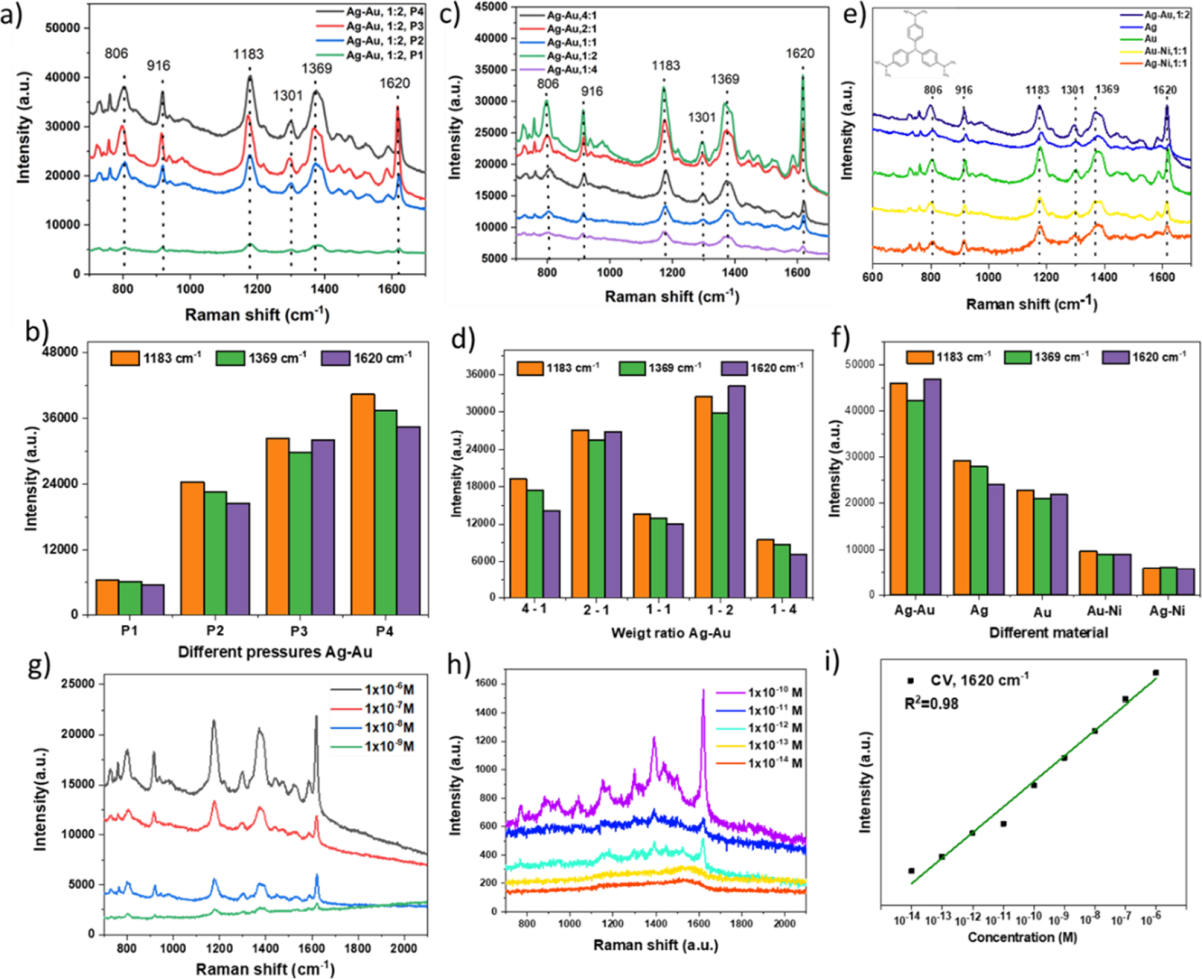
(a) SERS spectra of CV (10^–6^ M) recorded using Ag–Au NP films substrates obtained at varied pressures and (c) different Ag/Au ratios. (b, d) corresponding Raman intensities at three prominent peak positions of CV. (e) SERS spectra of CV (10^–6^ M) onto different obtained substrates. (f) corresponding SERS peak intensities at three different peak positions. (g, h) SERS spectra of CV with different concentrations onto the Ag–Au NP film substrate. (i) The linear calibration curve between SERS peak intensity at 1620 cm^–1^ vs. concentration of CV.

The SERS performance of the Ag–Au NP films was evaluated by functionalizing CV as a probe analyte molecule. As can be seen from Figure 4a,c, all the Ag–Au substrates showed a characteristic peak of CV at 806, 916, 1183, 1301, 1369, and 1620 cm^–1^ with different Raman peak intensities.^39^Figure 4a–d shows the SERS spectra of CV onto the Ag–Au NP films prepared at different pressures, Ag/Au ratios and associated SERS peak intensities at three different prominent peak positions of CV such as 1183, 1369, and 1620 cm^–1^.The optimization results showed that Ag–Au, 1:2, P4 film was found to exhibit higher SERS activities compared with the other substrates obtained. Notably, a slight red-shift in the peak positions of CV for the Ag–Au, 1:2, P3 sample was observed, which could be because of the large gaps in the self-assembled Ag–Au NP structure. It should be mentioned that the SERS signal intensity of CV over the Ag–Au, 1:2, P4 substrate is higher than that of the other substrates based on mono/bimetallic NP films obtained under the same conditions (Figure 4e,f). Moreover, the Ag–Au,1:2, P4 substrate also showed higher SERS activity with R6G as a probe molecule (Figure S5). The higher SERS signal intensities are attributed to the self-assembled 3D dendritic Ag–Au network with readily accessible hotspots between the gaps of the dendritic branches.

The EFs of the SERS substrate were estimated by following an earlier study.^40^ The Ag–Au,1:2, P4 substrate exhibited the highest SERS EFs, about ∼6.54 × 10^4^, which is higher than those of the other obtained SERS substrates (Figures S6 and S7). The estimated EF value was slightly lower compared with the calculated value by FDTD simulation, which could be a variation of the surface porosity involved in actual experiments. Furthermore, the SERS activity of the Ag–Au,1:2, P4 substrate is compared with SERS substrates prepared using the conventional chemical reduction method (Figure S8).^40^ The results showed that the Ag–Au,1:2, P4 substrate exhibits much higher SERS activity in detecting CV molecules compared with the SERS substrate obtained by the chemical reduction method, suggesting exceptional SERS performance. Given the above result, the Ag–Au, 1:2, P4 substrate is chosen for the subsequent SERS-detection experiments.

To evaluate the sensitivity of the SERS substrate (Ag–Au,1:2, P4), the SERS spectra were recorded by immobilizing different CV concentrations in the range between 10^–6^ and 10^–14^ M (Figure 4g–i)**.** The SERS peaks can be detected even at 10^–14^ M, indicating the low detection limit up to 10^–14^ M was reached. In addition to the high sensitivity, the substrate displayed excellent reproducibility of the Raman signal recorded from 25 randomly selected regions in the substrate functionalized with 10^–6^ M of CV molecules with a very low relative standard deviation (RSD) value of 5.8% (Figure S9), which is much lower compared to the commercial Au SERS substrates (∼20%). Furthermore, the SERS substrate exhibited acceptable sample-to-sample reliability with an RSD of 5.6% (Figure S10) and storage stability with only a 20% decrement for a 10-day period (Figure S11)**.**

### SERS Detection of PET Nanoplastic Particles

**Figure 5:**
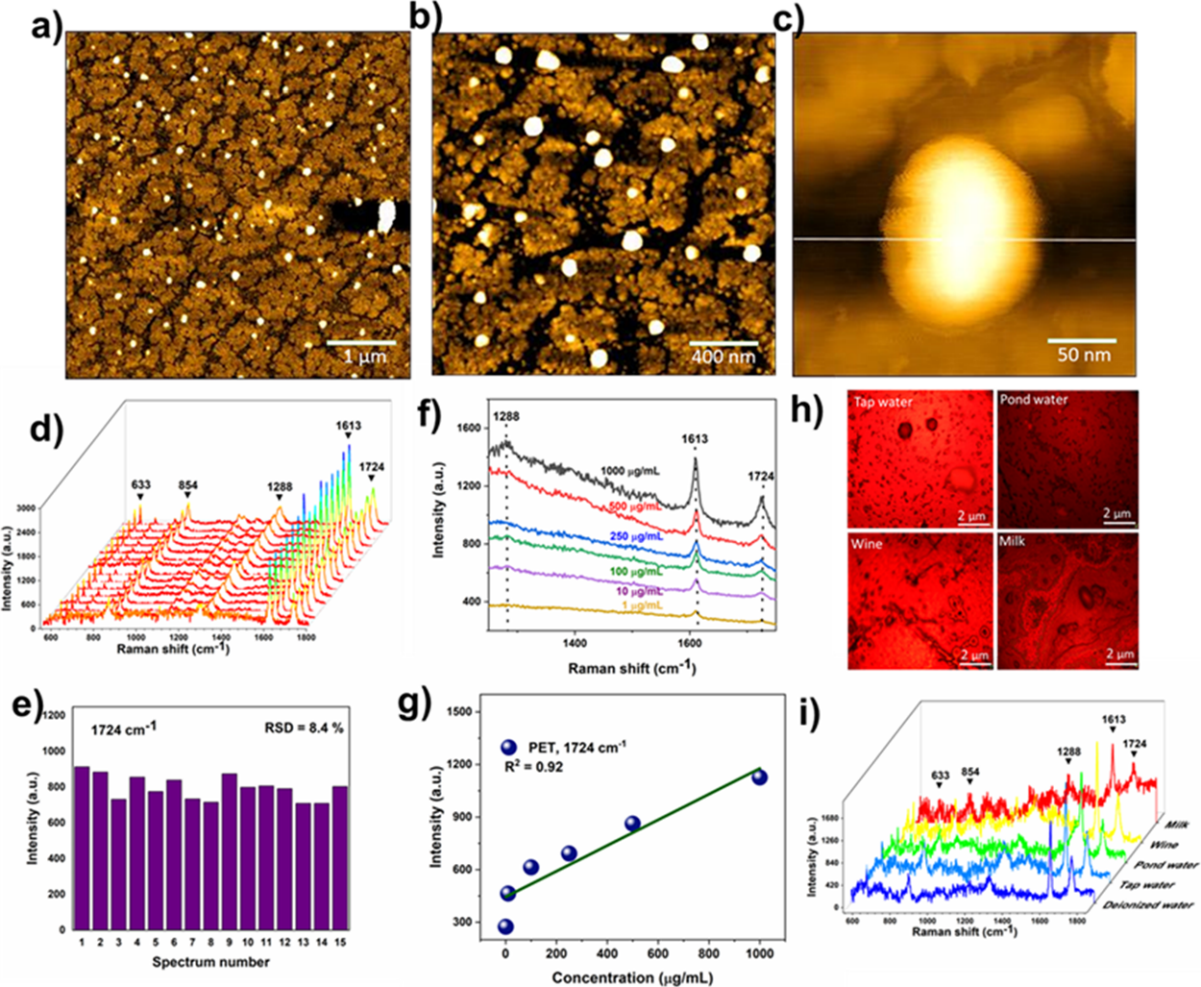
(a–c) AFM topological images. (d) SERS spectra of PET nanoplastics onto Ag–Au (1:2) P4 substrate collected from 15 different spots. (e) Corresponding Raman peak intensity at 1724 cm^–1^ for 15 spectra. (f) SERS spectra of PET nanoplastics with different concentrations. (g) Corresponding linear calibration plot. (h) Raman optical images of PET nanoplastics in four real samples. (i) SERS spectra of PET nanoplastics (100 mg/mL) in different real samples.

To demonstrate the practical applicability of the SERS-active substrate, we investigated SERS measurements for the detection of nanoplastic particles of polyethene terephthalate (PET) in water. Specifically, the PET nanoparticles (50–300 nm) (Figures S12 and S13) with varied concentrations were dispersed onto the Ag–Au, 1:2, P4 SERS substrate, and allowed to completely dry at ambient conditions and the SERS spectra were collected. As shown in Figure 5a–c, the AFM topographical images show the uniform distribution of PET nanoplastics over the substrate. Figure 5d shows the SERS spectra of PET nanoplastic particles over the Ag–Au NP substrate collected from 15 different spots in the same substrate exhibit five characteristic peaks at 633, 854, 1288, 1613, and 1724 cm^–1^, corresponding to the PET particles.^41^ Specifically, the peak at 633 cm^–1^ is associated with the vibration of aromatic C=C in-plane ring deformation, a peak at 855 cm^–1^ corresponds to the vibration of aromatic C=C out-of-plane deformation and a peak at 1288 cm^–1^ is assigned to the CH_2_ twisting vibrations of aromatic in-plane CH deformation.^42^ The characteristic peaks at 1613 and 1724 cm^–1^ are associated with the C–O and C=O stretching vibrations, respectively.^43^ The intensity of the peak at 1724 cm^–1^ remained almost the same for SERS spectra obtained from 15 different spots with a low RSD of 8.4%, suggesting excellent reproducibility (Figure 5e). As shown in Figure 5g,f, the Raman signals of PET nanoplastic were detected with concentrations as low as 1 μg/mL, which is higher related to recent reports (Table S2).^41,43−47^ Furthermore, the SERS substrate can detect commercial polystyrene nanospheres (PS, size ∼100 nm) with an LOD down to 0.1 mg/mL (Figure S14)**,** indicating that the proposed method enables monitoring another type of nanoplastics in real samples.

Detection of nanoplastics in real samples is crucial to their applications. Previous studies demonstrated the presence of nano/microplastics with a size ranging between 5 mm and 700 nm in a variety of environmental, food, and blood samples.^48,49^ Therefore, we have evaluated its analytical ability by detecting PET nanoplastics in tap water, pond water, diluted milk, and wine samples. As shown in Figure 5, h,i, the Raman signals detected PET nanoplastics with a concentration as low as 100 mg/mL in four different real samples. Notably, no new peaks are detected in the four real samples without dispersion of PET nanoplastics (Figure S15), suggesting there are no other types of plastics present or the presence of very low concentrations, which exceeds the ability of detection in the proposed method.

### Detection of PET Particles in Commercial Drinking Water Bottles

**Figure 6:**
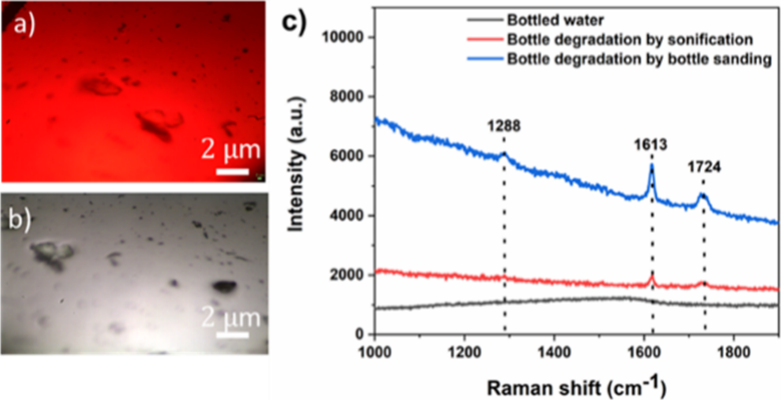
(a,b) Raman optical images. (c) SERS spectra of the PET micro/nanoplastics extracted from the drinking water bottle onto the Ag–Au NP SERS substrate.

To further demonstrate the practical application, we have investigated SERS-detection PET by degradation of nanoplastics in commercially available drinking water bottles. Figure 6a,b shows the Raman optical images of the PET nanoplastic degraded from the bottled drinking water, revealing that the small irregular PET particles were deposited on the Ag–Au NP film substrate. As shown in Figure 6c, the Raman spectra showed a characteristic peak at 1613, 1724, and 1288 cm^–1^, which corresponds to the C–O stretching and C=O stretching, and CH_2_ twisting vibrations of aromatic in-plane CH deformation peaks of PET nanoplastics.^50^ However, no new distinguishable peaks were observed in blank water sample bottles without a degradation process. These results demonstrate that the proposed detection method has great potential for the analysis of nano/microplastics in the actual water environment.

## Conclusions

In summary, we demonstrated a simple, effective method based on a DES-assisted vacuum thermal evaporation technique for fabricating a 3D self-assembly network of Ag–Au NP films with well defined nanometric gaps. Increasing the vacuum thermal evaporation pressure and optimizing the Ag/Au ratio enabled the achievement of controlled self-assembly of Ag–Au NPs with tunable shapes and gaps within the close-packed structure. The fabricated SERS substrate using dendritic Ag–Au NP film exhibited superior SERS activity with a LOD of 10^–14^ M of CV analyte molecules, as well as excellent reproducibility, and stability. In addition, the substrate exhibited superior sensitivity for the detection of PET nanoplastics, with an LOD down to 1 μg/mL. Finally, the SERS substrate demonstrated that the capability of detecting PET nanoplastics with excellent sensitivity in real environmental samples as well as actual commercial drinking water bottles containing PET nanoplastics provides a potential opportunity in various SERS applications.

## Supporting Information Available

Experimental methods, SEM images of large area self-assembled Ag–Au NP films, SEM images of Au, Ag, Ag–Ni, and Au–Ni NP films SERS spectra of R6G immobilized Ag–Au substrate, SEM, AFM images of PET nanoplastics, SERS detection of PS nanoplastics (PDF)
