# Supplementary material for: Ionic Liquid-Assisted Thermal Evaporation of Bimetallic Ag–Au Nanoparticle Films as a Highly Reproducible SERS Substrate for Sensitive Nanoplastic Detection in Complex Environments
Source: Anal Chem. 2024 Mar 7;96(15):5790–7. doi: 10.1021/acs.analchem.3c04442 (PMC11024884; doi:10.1021/acs.analchem.3c04442)
Supplement: Supplementary file 1 — ac3c04442_si_001.pdf [file ac3c04442_si_001.pdf]

Supporting information:

Ionic Liquid-Assisted Thermal Evaporation of Bimetallic Ag-Au Nanoparticle Films as a Highly Reproducible SERS Substrate for Sensitive Nanoplastic Detection in Complex Environments

Rafael V. Carreón, † Orlando C. Martínez, ‡ Ana G. Rodríguez-Hernández, # Laura E. Serrano de la Rosa, $ J.J. Gervacio-Arciniega,ᵠ * Siva Kumar Krishnan.§*

†Facultad de Ciencias Físico Matemáticas, Benemérita Universidad Autónoma de Puebla, Av. San Claudio y Av. 18 sur., Puebla, Pue., C. P. 72570, México.

‡CINVESTAV-Unidad Querétaro, Libramiento Norponiente No. 2000, Real de Juriquilla, Querétaro, Qro. 76230, México.

CONAHCyT-Centro de Nanociencias and Nanotecnología, Universidad Nacional Autónoma de México, Km 107 Carretera Tijuana-Ensenada Apdo Postal 14, CP. 22800 Ensenada, B.C. México

$Instituto de Física, Benemérita Universidad Autónoma de Puebla, Apdo. Postal J-48, Puebla, Pue. 72570, México.

ᵠCONAHCyT- Facultad de Ciencias Físico Matemáticas, Benemérita Universidad Autónoma de Puebla, Apdo. Postal J-48, Puebla 72570, México.

§CONAHCyT-Instituto de Física, Benemérita Universidad Autónoma de Puebla, Apdo. Postal J-48, Puebla, Pue. 72570, México.

Corresponding authors:

JJG: jjgervacio@hotmail.com

SKK: sivakumar@ifuap.buap.mx

Table of Content

Figure S1: Typical low magnification SEM image of self-assembled dendritic like Ag-Au nanostructures films onto glass substrate. Deposition pressure of 2x10^{-4} mbar. ……………………………………………..**S4**

Figure S2. a, b) Typical SEM images of Ag-Au films obtained without utilizing DES onto growth substrate (glass). Deposition pressure of 2x10^{-4} mbar, applied current of 4 amperes………………………………. **S5**

Figure S3. Typical SEM images of Ag-Au NPs films were deposited onto different types of DESs. a, b) ChCl: Urea (ratio of 1:2), c, d) ChCl: malonic acid (molar ratio of 1:2), and e, f) ChCl: Ethylene glycol (Molar ratio of 1:4), respectively. All the films were deposited using Ag-Au ratio of 1:2 wt% at deposition pressure of 2x10^{-4} mbar…………………………………………………………………………………………... **S6**

Figure S4: SEM and energy dispersive spectroscopy (EDS)-elemental mapping of Ag-Au NPs films of three different composition. a-c) Ag-Au (1:4), d-f) Ag-Au (1:2) and g-i) Ag-Au (4:1), respectively…………….. **S7**

Figure S5. Typical SERS spectra of Rhodamine 6G (R6G) onto Ag-Au SERS substrates. a) SERS spectra of R6G onto Ag-Au on glass substrate obtained at different thermal evaporation pressures b) corresponding SERS peak intensities at 1312, 1365, and 1510 cm^{-1}, respectively. c) SERS spectra of R6G onto Ag-Au NPs films obtained at varied Ag/Au ratios, and d) corresponding peak intensities of peaks at 1312, 1365, and 1510 cm^{-1}, respectively…………………………………………………………………………………………**S8**

Figure S6. Comparison of the SERS enhancement factors value for different substrates. a) Ag-Au NPs film substrates obtained by varying the deposition pressure b) Ag-Au NPs films obtained by varying Ag/Au ratio. **………………………………………………………………………………………...S9**

Figure S7. A) SEM image of a) Ag, b) Au, c) Ag-Ni, and d) Au-Ni nanoparticle films deposited on glass substrate under pressure of 2x10^{-4} mbar B) Comparison of the SERS EFs of self-assembled Ag-Au NPs films substrates with EFs values of different substrate obtained under similar conditions……………………………………………………………………………………….. **S10**

Figure S8: a) UV-vis spectra of Au and Au-Ag NPs. b, c, d) STEM images of Au NPs, Au-Ag NPs, and Au-Ag nanocubes, e) comparison of SERS spectra of CV onto the Au NPs, Au-Ag NPs and Au-Ag nanocubes. D) corresponding Raman peak intensity of CV at 1620 cm^{-1} for different SERS substrates………………….. **S11**

Figure S9. SERS spectra of CV (1x10^{-6} M) on 25 different sports in the same substrate of Ag-Au (1:2) p4 substrates, b) corresponding signal intensities of peak at 1620 cm^{-1} vs number of spots…………………. **S13**

Figure S10. SERS spectra of CV (1x10^{-6}M) on six-different self-assembled Ag-Au film substrates, b) corresponding signal intensities of peak at 1620 cm^{-1} vs six-substrates…………………………………. **S14**

Figure S11. Stability of the self-assembled Ag-Au film substrates. a) SERS spectra of CV (10^{-6} M) covered on self-assembled Ag-Au SERS substrate collected in each 2-days for 10 days period. B) The variation of SERS peak intensity at 120 cm^{-1} as a function of storage time (days)…………………………………………. **S15**

Figure S12. a) TEM image, b) dynamic light scattering (DLS) histogram showing size distribution of PET nanoplastic particles……………………………………………………………………………. **..S16**

Figure S13. a) AFM topographical images of PET nanoplastic spheres onto the Ag-Au NPs film substrate, b) AFM height profile analysis of PET particles over the substrate………………………………………. **.S17**

Figure S14: a, b) AFM images of polystyrene nanospheres (size=100 nm) onto self-assembled Ag-Au NPs film substrate. c SERS spectra of PS nanospheres with different concentration over Ag-Au NPs film substrate, d)

corresponding linear calibration plot of Raman intensity at 1001 cm-1 vs. PS nanosphere concentration. e) Raman images of PS nanospheres containing real samples, f) SERS spectra of PS nanospheres containing real-samples…………………………………………………………………………………………………….. **S19**

Figure 15: SERS spectra of real samples (tap, pond water, diluted milk and wine) deposited on the Ag-Au,1:2, P4 NPs films substrate**……………………………………………………………………………………… S20**

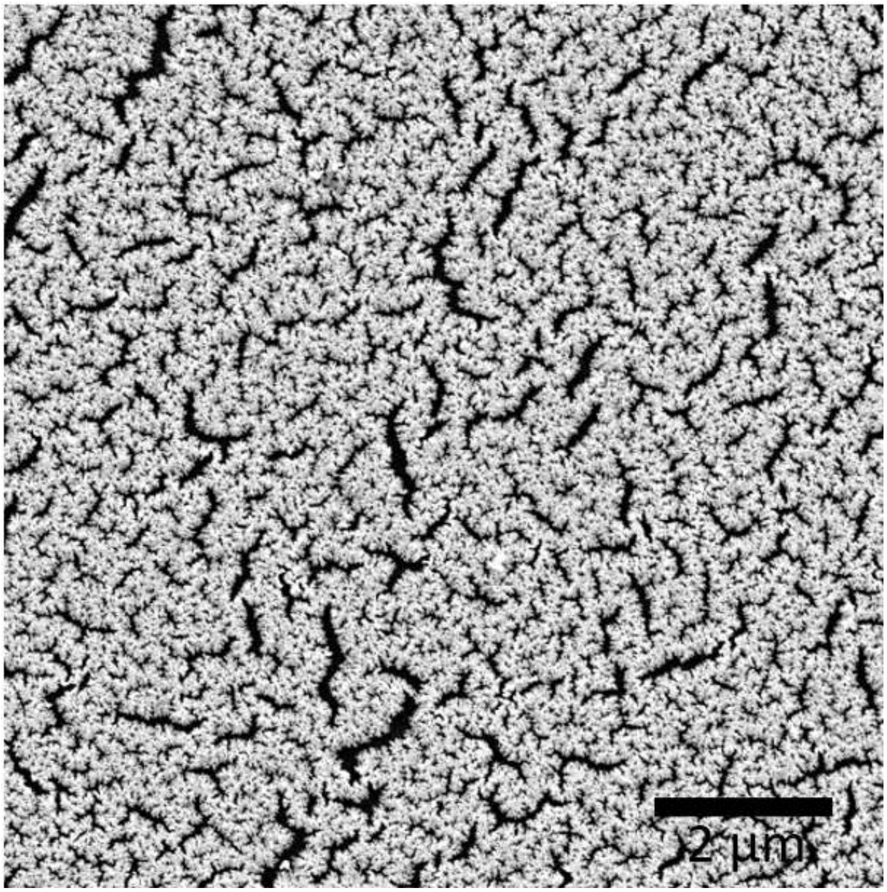

Figure S-1. Typical low magnification SEM image of self-assembled dendritic like Ag-Au nanostructures films onto glass substrate. Deposition pressure of 2x10^{-4} mbar.

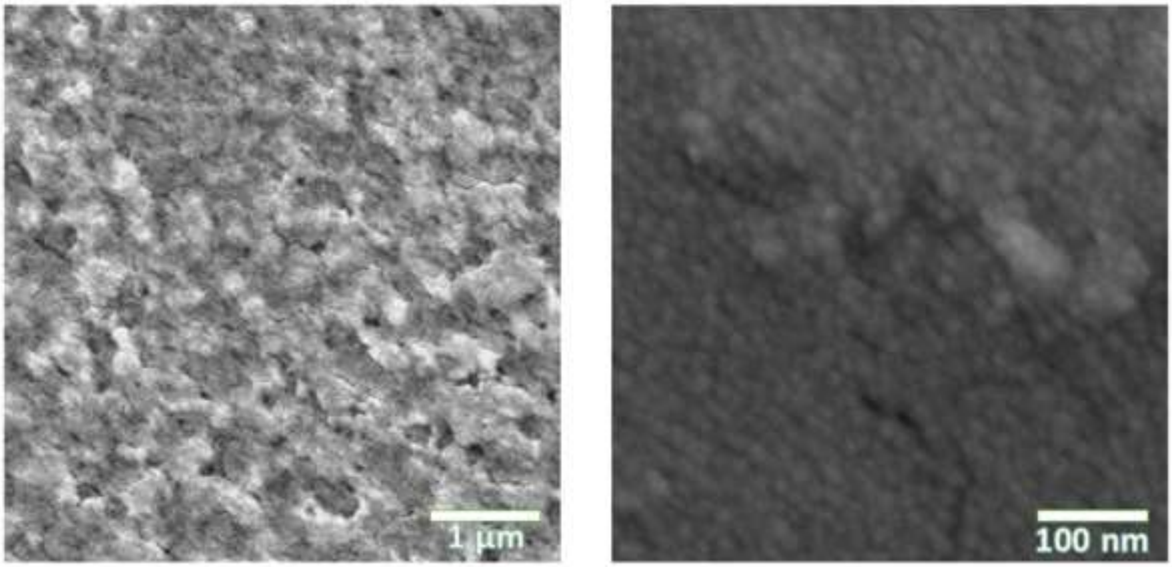

Figure S2. a, b) Typical SEM images of Ag-Au films obtained without utilizing DES onto growth substrate (glass). Deposition pressure of $2x10^{-4}$ mbar, applied current of 4 amperes.

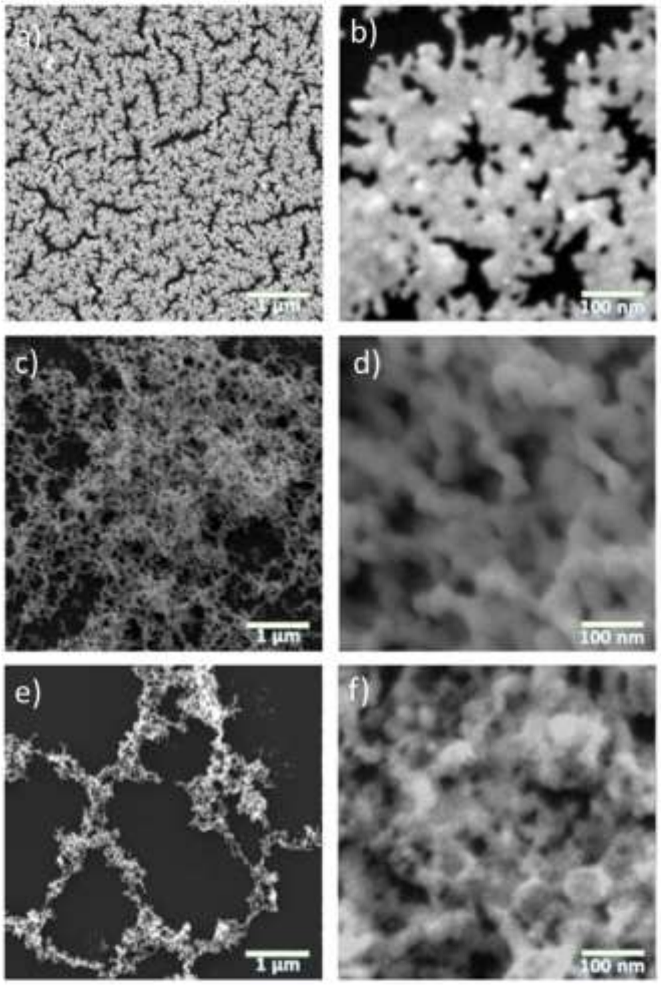

Figure S3. Typical SEM images of Ag-Au NPs films were deposited onto different types of DESs. a, b) ChCl: Urea (ratio of 1:2), c, d) ChCl: malonic acid (molar ratio of 1:2), and e, f) ChCl: Ethylene glycol (Molar ratio of 1:4), respectively. All the films were deposited using Ag-Au ratio of 1:2 wt% at deposition pressure of $2x10^{-4}$ mbar.

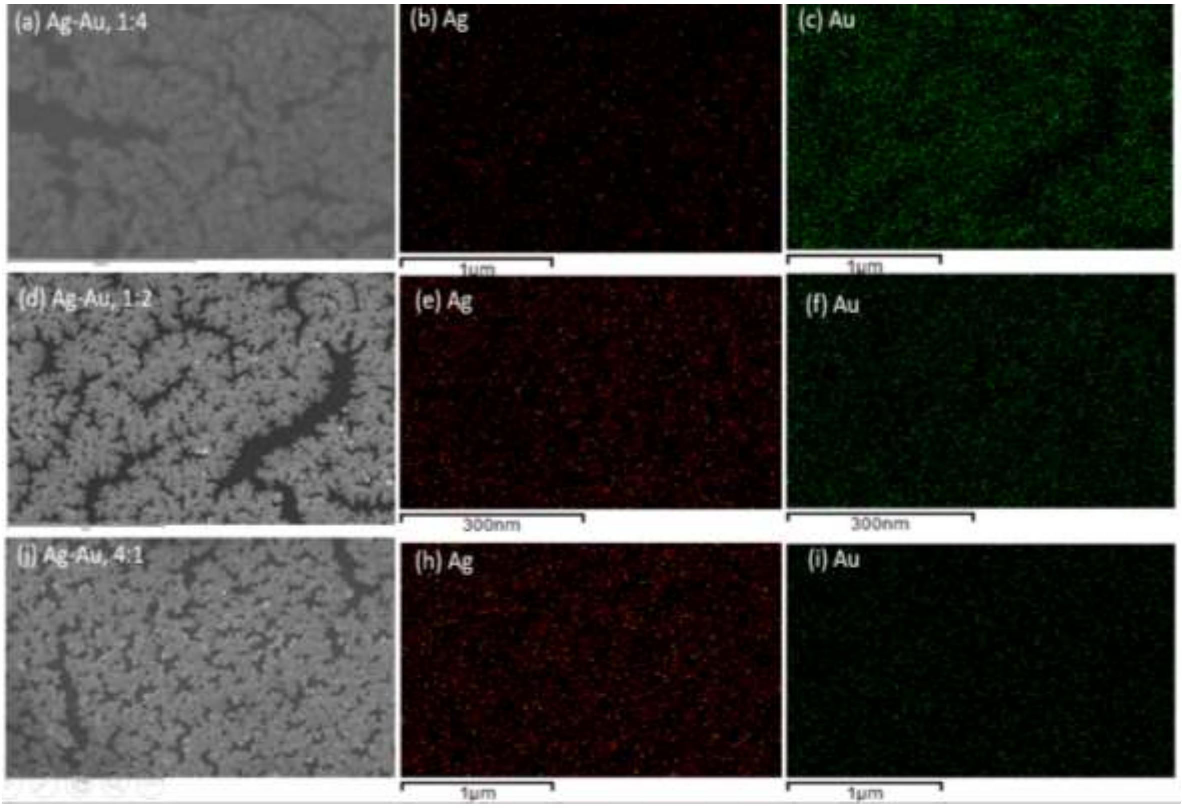

Figure S4: SEM and energy dispersive spectroscopy (EDS)-elemental mapping of Ag-Au NPs films of three different composition. a-c) Ag-Au (1:4), d-f) Ag-Au (1:2) and g-i) Ag-Au (4:1), respectively.

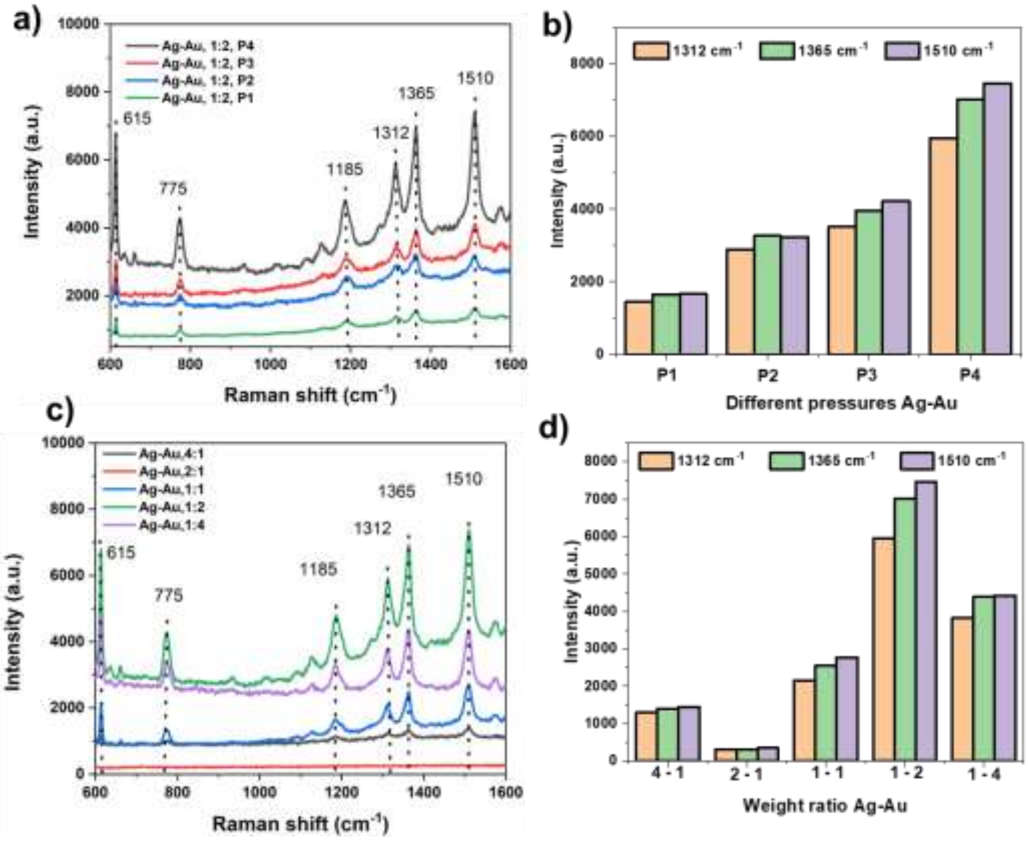

Figure S5. Typical SERS spectra of Rhodamine 6G (R6G) onto Ag-Au SERS substrates. a) SERS spectra of R6G onto Ag-Au on glass substrate obtained at different thermal evaporation pressures b) corresponding SERS peak intensities at 1312, 1365, and 1510 cm^{-1}, respectively. c) SERS spectra of R6G onto Ag-Au NPs films obtained at varied Ag/Au ratios, and d) corresponding peak intensities of peaks at 1312, 1365, and 1510 cm^{-1}, respectively.

SERS Enhance Factor (EF) estimation:

The estimation of SERS enhancement factor (EF) was estimated based on following the previous studies.[1] The SERS EFs for the CV covered substrates were calculated using the following relation:

$$EF = \frac{I_{SERS}}{N_{SERS}} \Big/ \frac{I_{Nor}}{N_{Nor}} \qquad \text{-----------(1)}$$

Where, I_{SERS} and I_{Nor} are the signal intensities of SERS and normal Raman spectra of CV for the same dispersion band (1620 cm^{-1}), and N_{SERS} and N_{Nor} represent the corresponding number of molecules in the focused incident laser spot. Assuming a uniform distribution of CV molecules over the substrates, the values of N_{SERS} and N_{Nor} in eq.1 can be substituted by the concentration of CV, that is, 1.0×10^{-6} and 1.0×10^{-3} M, respectively.

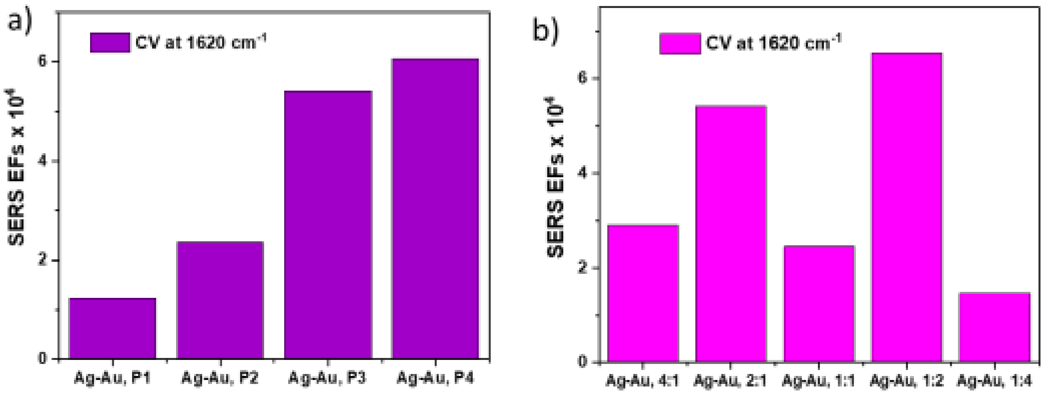

Figure S6. Comparison of the SERS enhancement factors values for different substrates. a) Ag-Au NPs film substrates obtained by varying the deposition pressure b) Ag-Au NPs films obtained by varying Ag/Au ratio.

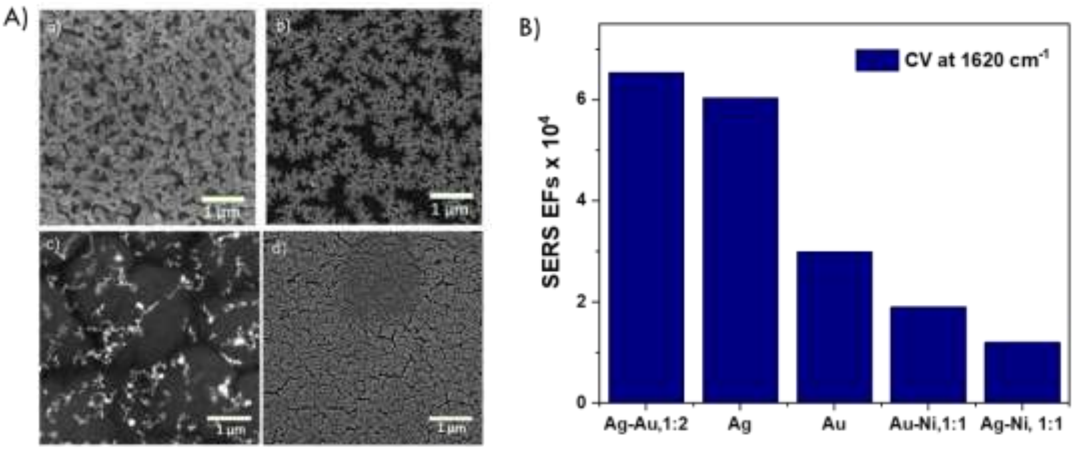

Figure S7. A) SEM images of a) Ag, b) Au, c) Ag-Ni, and d) Au-Ni nanoparticle films deposited on glass substrate under pressure of $2x10^{-4}$ mbar. B) Comparison of the SERS EFs of self-assembled Ag-Au NPs films substrates with EFs values of different substrate obtained under similar conditions.

Chemical Synthesis of gold nanoparticles and Au-Ag bimetallic nanoparticles and SERS Analysis:

The spherical Au NPs with particle sizes of approximately 10 nm, and Au-Ag core-shell NPs and nanocubes were synthesized by following earlier report.[2] The formation of spherical Au NPs, Au-Ag, and Ag-Au core-shell NPs was confirmed using UV-vis and TEM imaging (Figure S8). Then, 50 µL of cleaned colloidal dispersion was deposited over glass substrate through drop casting, and allowed to dry to obtain SERS substrate based on Au NPs and Au-Ag NCs. After that, 20 µL of CV (10-6 M) was deposited over the substrate and SERS spectra were recorded.

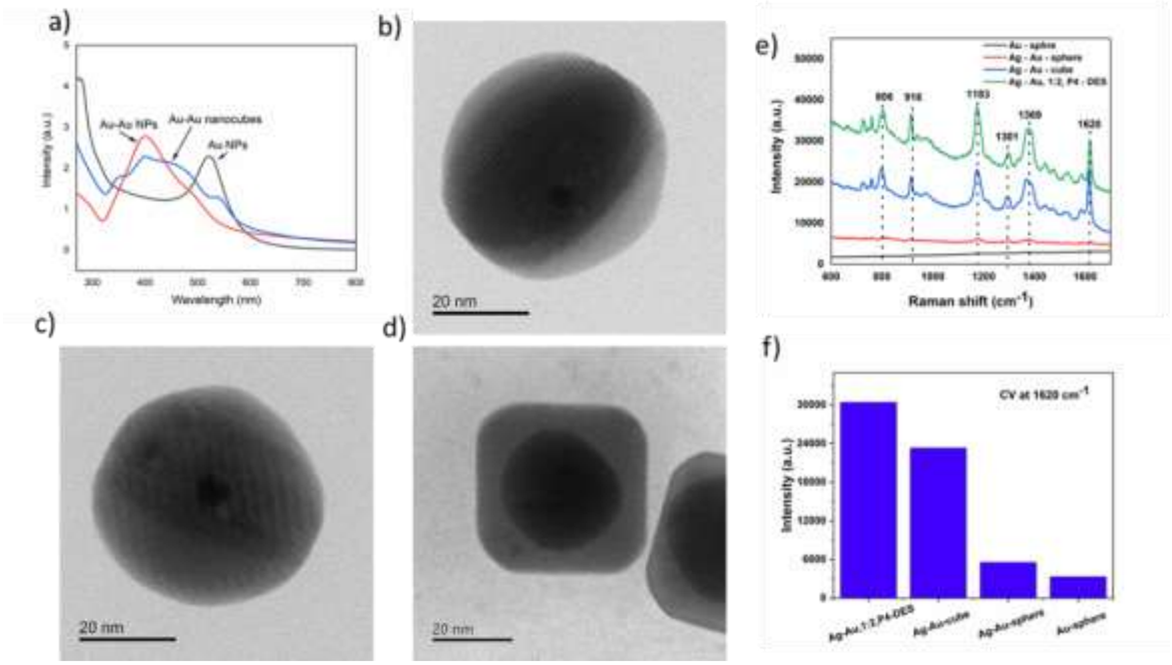

Figure S8: a) UV-vis spectra of Au and Au-Ag NPs. b, c, d) STEM images of Au NPs, Au-Ag NPs, and Au-Ag nanocubes, e) comparison of SERS spectra of CV onto the Au NPs, Au-Ag NPs and Au-Ag nanocubes. D) corresponding Raman peak intensity of CV at 1620 cm^{-1} for different SERS substrates.

Table S1: Comparison of SERS detection performance of Ag-Au NPs film substrate with the previous reports.

SERS substrate	Analyte molecules	Wavelength of laser excitation	EFs	LOD (M)	Signal uniformity	Reference
Au-Ag alloy Patterned nanostructures	Malachite green dye		1.09 $\times 10^{9}$	$1x10^{-14}$	< 6.5 ± 0.3%	Lee et al.[3]
Porous Au nanoparticles arrays	Rhodamine 6G		$1.4x10^{7}$	10-11	6.6%	Liu et al.,[4]
Porous Ag films	Rhodamine 6G		4.2×10^{10}	$1x10^{-13}$		Dou et al.[5]
Porous Ag-Au nanoparticles array	Rhodamine 6G	532	$2.2x10^{7}$	10^{-9}	7.7%	Li et.al.,[6]
Micropyramid Array	Rhodamine 6G		8.8×10^{9}	$1x10^{-14}$	4.99	Zhang et al.[7]
Porous Ag-Au/silica nanostructues	CV	633	$1.3x10^{7}$	NA	NA	Liu et al.,[8]
Au-Cu nanoflower	CV	633	$0.21x10^{6}$	10^{-10}	13.8%	Siva Kumar et al.,[1]
Ag-Au Nanostar	CV		$1x10^{7}$	$0.015x10^{-9}$	NA	Li et al.,[9]

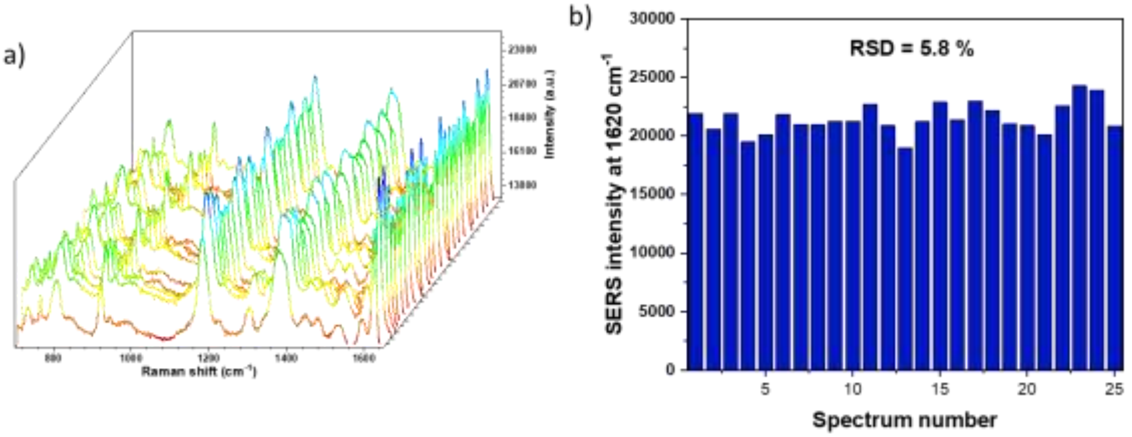

Figure S9. SERS spectra of CV (1x10^{-6} M) on 25 different sports in the same substrate of Ag-Au (1:2) p4 substrates, b) corresponding signal intensities of peak at 1620 cm^{-1} vs number of spots.

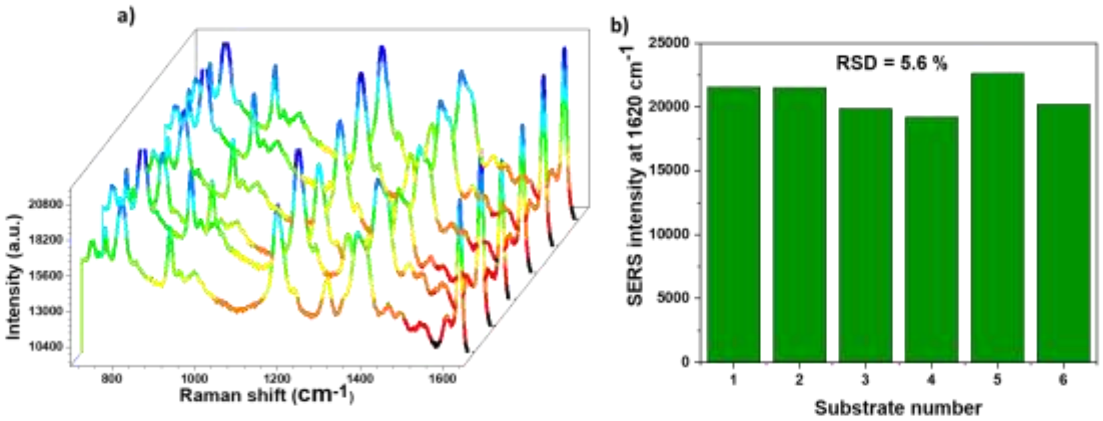

Figure S10. SERS spectra of CV ($1x10^{-6}$ M) on six-different self-assembled Ag-Au film substrates, b) corresponding signal intensities of peak at 1620 cm^{-1} vs six-substrates.

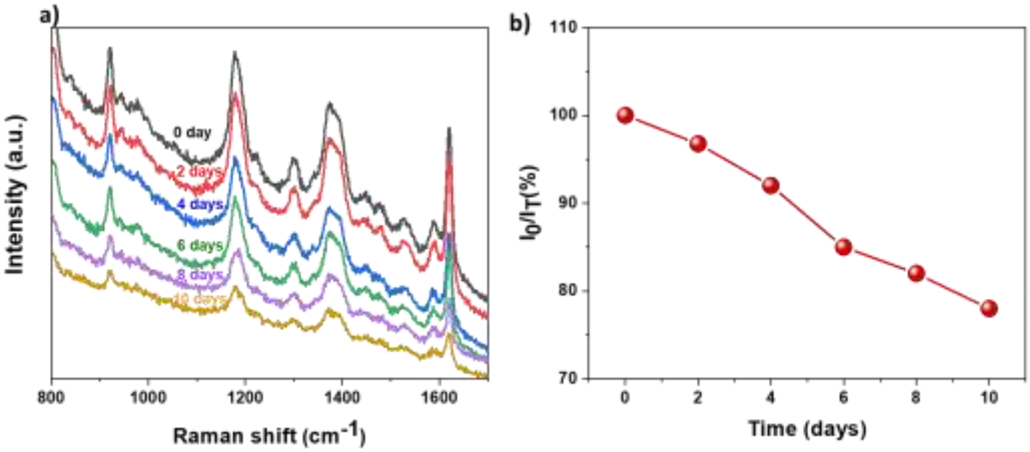

Figure S11. Stability of the self-assembled Ag-Au film substrates. a) SERS spectra of CV (10^{-6} M) covered on self-assembled Ag-Au SERS substrate collected in each 2-days for 10 days period. b) The variation of SERS peak intensity at 120 cm^{-1} as a function of storage time (days).

PET Nanoplastic synthesis and characterization.

The PET nanoplastics were synthesized by using our previously reported protocol. Ref Specifically, 1 gram of PET nanoplastic particles (size 50-300 nm) was dissolved in 10 mL of concentrated trifluoroacetic acid solution (TFA, 90% v/v) at 50°C and stirred for 2 hours until complete dissolution. After that, the solution was kept overnight. To obtain nanoplastic particles, the above solution was precipitated by adding 10 mL of diluted TFA (20% v/v) under vigorous stirring and kept for 2 hrs. The suspension was centrifuged at 2500 rpm for 1 hr to separate the nanoplastic particles.

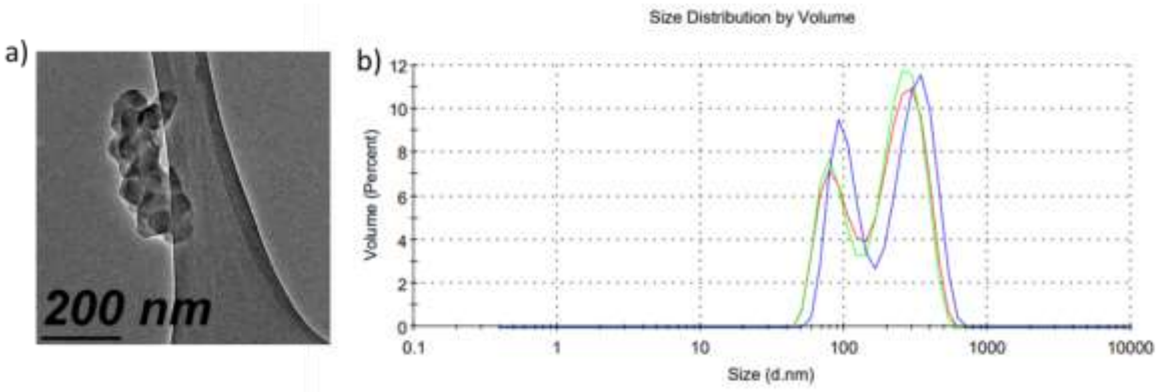

Figure S12. a) TEM image, b) dynamic light scattering (DLS) histogram showing size distribution of PET nanoplastic particles.

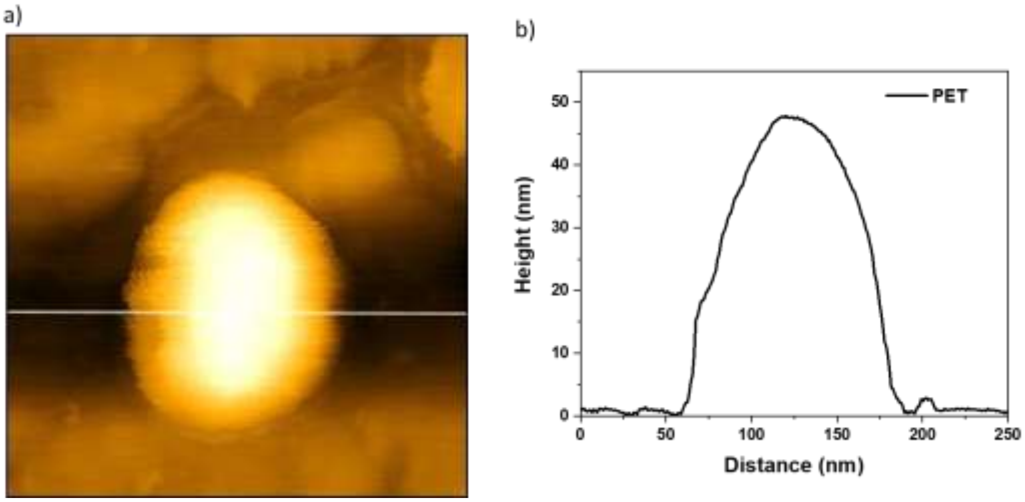

Figure S13. a) AFM topographical images of PET nanoplastic spheres onto the Ag-Au NPs film substrate, b) AFM height profile analysis of PET particles over the substrate.

Table S2. Comparison of the SERS-detection parameters for Ag-Au NPs films substrate with recently reported SERS substrates for nanoplastic detection

SERS substrate	Type of micro/nano plastic particles	Micro/nanoplastic particles size	Sensitivity	References
Ag/ZnO@PDMS	Polystyrene	800 nm	25 µg/mL	Zhu et al.,[10]
Au NPs	Polystyrene	1-4 µm	6.5 µg/mL	Mikac et al.[11]
Au pyramidal cavities	Polystyrene<br>PMMA	360 nm<br>500 nm	26.3 µg/mL<br>26.3 µg/mL	Xu et al.,[12]
Au NPs decorated sponge	4-mercaptopyridine (4-MPY)	39-155 µm	50 µg/mL	Yin et al.[13]
AgNPs− $MgSO_4$	Polystyrene	50 nm, 1 µm	100, 100 µg/mL	Zhou et al.[14]
AuNSs@Ag@AAO	Polystyrene	400 nm	50 µg/mL	Le tal.[15]
Au triangular cavity array	Poly(ethylene terephthalate)	88.2 nm	10^8 particles/mL	Zhang et al.[16]
Self-assembled Ag-Au NPs film	Poly(ethylene terephthalate)	50-300 nm	1 µg/mL	this work

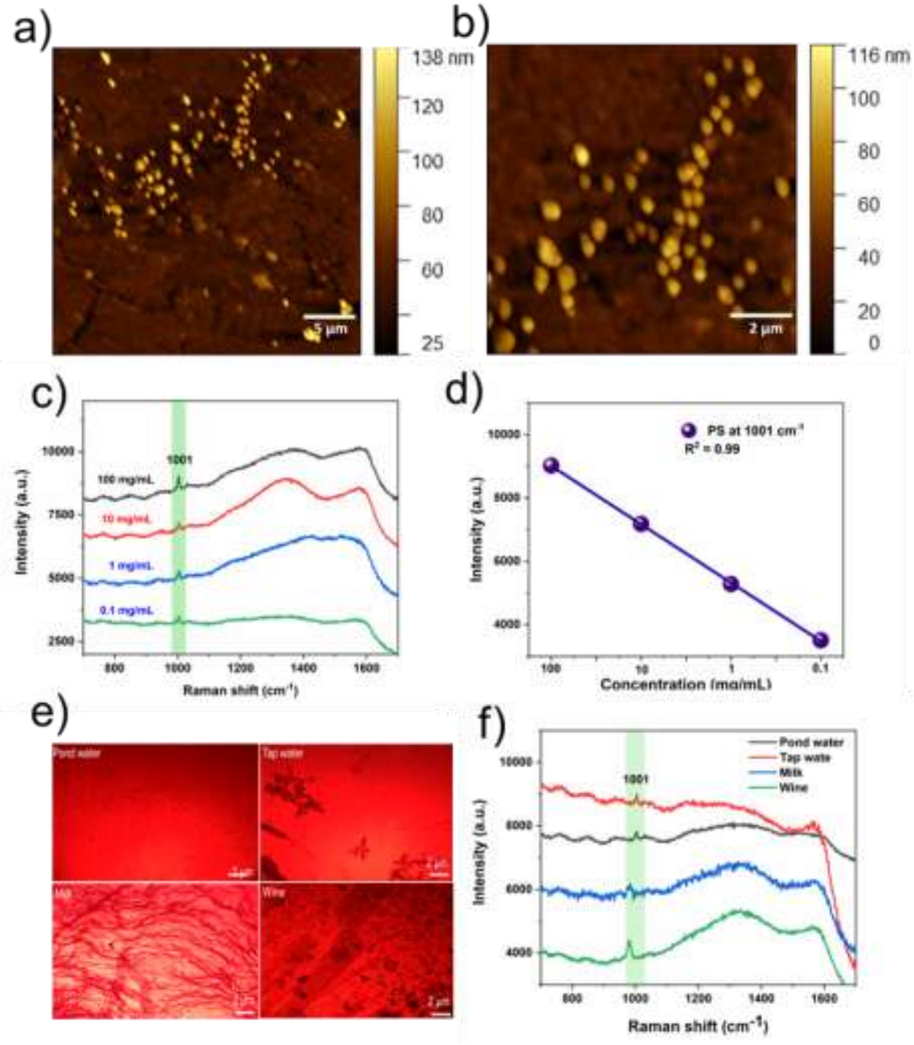

Figure S14: a, b) AFM images of polystyrene nanospheres (size=100 nm) onto self-assembled Ag-Au NPs film substrate. c) SERS spectra of PS nanospheres with different concentration over Ag-Au NPs film substrate, d) corresponding linear calibration plot of Raman intensity at 1001 cm^{-1} vs. PS nanosphere concentration. e) Raman images of PS nanospheres containing real-samples, f) SERS spectra of PS nanospheres (10 mg/mL) containing real-samples.

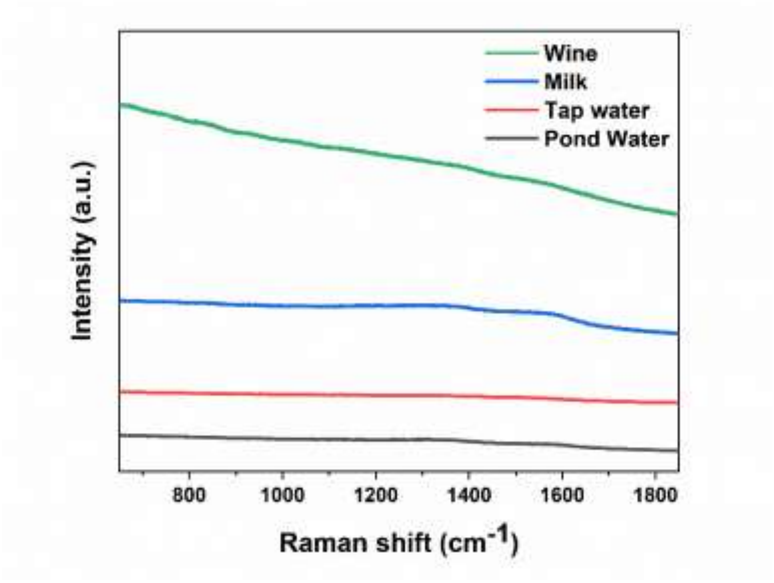

Figure S15: SERS spectra of real samples (tap, pond water, diluted milk and wine) deposited on the Ag-Au,1:2, P4 NPs films substrate**.**

PET nanoparticles formation in real-water samples:

To obtain PET micro/nanoparticles, the commercial water bottle was degraded using 2 methods. In the first method, a bottle was placed in water heated to 80°C and kept in an ultrasonic bath for 5 hours. After that time, 50 μL PET containing water were taken from the bottle and were deposited on the Ag-Au 1:2 P4 substrate. In the second method, the upper part of the commercial water bottle was sanded for 30s, the particles obtained were dispersed in 20 mL of deionized water and they were introduced into an ultrasonic bath for 1 hour. Finally, 50 μL of the prepared solution was taken and deposited on the Ag-Au-1:2-P4 substrate, all samples were left to dry at room temperature.

References:

(1) Kumar-Krishnan, S.; Esparza, R.; Pal, U. Controlled Fabrication of Flower-Shaped Au-Cu Nanostructures Using a Deep Eutectic Solvent and Their Performance in Surface-Enhanced Raman Scattering-Based Molecular Sensing. *ACS Omega* **2020**, *5*, 3699–3708

(2) Steiner, A. M.; Mayer, M.; Schletz, D.; Wolf, D.; Formanek, P.; Hübner, R.; Dulle, M.; Förster, S.; König, T. A. F.; Fery, A. Silver Particles with Rhombicuboctahedral Shape and Effective Isotropic Interactions with Light. *Chemistry of Materials* **2019**, *31*, 2822–2827.

(3) Lee, T.; Kwon, S.; Lee, J. J. Highly Dense and Accessible Nanogaps in Au-Ag Alloy Patterned Nanostructures for Surface-Enhanced Raman Spectroscopy Analysis. *ACS Appl Nano Mater* **2020**, *3*, 5920–5927

(4) Liu, G.; Li, K.; Zhang, Y.; Du, J.; Ghafoor, S.; Lu, Y. A Facile Periodic Porous Au Nanoparticle Array with High-Density and Built-in Hotspots for SERS Analysis. *Appl Surf Sci* **2020**, *527*, 146807.

(5) Dou, Z.; Zhao, Z.; Zhang, M.; Xie, Y.; Yu, W.; Chen, Y. Uniform Near-Spherical Nanoscale Silver Films for Surface-Enhanced Raman Spectroscopy Sensing. *ACS Appl Nano Mater* **2020**, *3*, 2008–2015.

(6) Li, K.; Liu, G.; Zhang, S.; Dai, Y.; Ghafoor, S.; Huang, W.; Zu, Z.; Lu, Y. A Porous Au-Ag Hybrid Nanoparticle Array with Broadband Absorption and High-Density Hotspots for Stable SERS Analysis. *Nanoscale* **2019**, *11*), 9587–9592.

(7) Zhang, C.; Chen, S.; Jiang, Z.; Shi, Z.; Wang, J.; Du, L. Highly Sensitive and Reproducible SERS Substrates Based on Ordered Micropyramid Array and Silver Nanoparticles. *ACS Appl Mater Interfaces* **2021**, *13*, 29222–29229.

(8) Liu, K.; Bai, Y.; Zhang, L.; Yang, Z.; Fan, Q.; Zheng, H.; Yin, Y.; Gao, C. Porous Au-Ag Nanospheres with High-Density and Highly Accessible Hotspots for SERS Analysis. *Nano Lett* **2016**, *16*, 3675–3681.

(9) Li, Y. Le; Zhu, J.; Weng, G. J.; Liu, Y. N.; Li, J. J.; Zhao, J. W. Study on the Roughen Process of Branches of AuAg Nanostars for the Improved Surface-Enhanced Raman

Scattering (SERS) to Detect Crystal Violet in Fish. *Sens Actuators B Chem* **2023**, *390*, 133936.

(10) Zhu, Z.; Han, K.; Feng, Y.; Li, Z.; Zhang, A.; Wang, T.; Zhang, M.; Zhang, W. Biomimetic Ag/ZnO@PDMS Hybrid Nanorod Array-Mediated Photo-Induced Enhanced Raman Spectroscopy Sensor for Quantitative and Visualized Analysis of Microplastics. *ACS Appl Mater Interfaces* **2023**, *15*, 36988–36998.

(11) Mikac, L.; Rigó, I.; Himics, L.; Tolić, A.; Ivanda, M.; Veres, M. Surface-Enhanced Raman Spectroscopy for the Detection of Microplastics. *Appl Surf Sci* **2023**, *608*, 155239-155248.

(12) Xu, G.; Cheng, H.; Jones, R.; Feng, Y.; Gong, K.; Li, K.; Fang, X.; Tahir, M. A.; Valev, V. K.; Zhang, L. Surface-Enhanced Raman Spectroscopy Facilitates the Detection of Microplastics <1 Mm in the Environment. *Environ Sci Technol* **2020**, *54* (24), 15594–15603.

(13) Yin, R.; Ge, H.; Chen, H.; Du, J.; Sun, Z.; Tan, H.; Wang, S. Sensitive and Rapid Detection of Trace Microplastics Concentrated through Au-Nanoparticle-Decorated Sponge on the Basis of Surface-Enhanced Raman Spectroscopy. *Environmental Advances* **2021**, *5*,100096-100104.

(14) Zhou, X. X.; Liu, R.; Hao, L. T.; Liu, J. F. Identification of Polystyrene Nanoplastics Using Surface Enhanced Raman Spectroscopy. *Talanta* **2021**, *221*.

(15) Lê, Q. T.; Ly, N. H.; Kim, M. K.; Lim, S. H.; Son, S. J.; Zoh, K. D.; Joo, S. W. Nanostructured Raman Substrates for the Sensitive Detection of Submicrometer-Sized Plastic Pollutants in Water. *J Hazard Mater* **2021**, *402*.

(16) Zhang, J.; Peng, M.; Lian, E.; Xia, L.; Asimakopoulos, A. G.; Luo, S.; Wang, L. Identification of Poly(Ethylene Terephthalate) Nanoplastics in Commercially Bottled Drinking Water Using Surface-Enhanced Raman Spectroscopy. *Environ Sci Technol* **2023**, *57*, 8365–8372.
